# Intra-Subject Consistency during Locomotion: Similarity in Shared and Subject-Specific Muscle Synergies

**DOI:** 10.3389/fnhum.2017.00586

**Published:** 2017-12-04

**Authors:** Daniele Rimini, Valentina Agostini, Marco Knaflitz

**Affiliations:** Dipartimento di Elettronica e Telecomunicazioni, Politecnico di Torino, Turin, Italy

**Keywords:** muscle synergies, electromyography, locomotion, gait analysis, repeatability

## Abstract

Human locomotion is a complex motor task. Previous research hypothesized that muscle synergies reflect the modular control of muscle groups operated by the Central Nervous System (CNS). Despite the high stride-to-stride variability characterizing human gait, most studies analyze only a few strides. This may be limiting, because the intra-subject variability of motor output is neglected. This gap could be filled by recording and analyzing many gait cycles during a single walking task. In this way, it can be investigated if CNS recruits the same muscle synergies consistently or if different strategies are adopted during the locomotion task. The aim of this work is to investigate the intra-subject consistency of muscle synergies during overground walking. Twelve young healthy volunteers were instructed to walk for 5 min at their natural pace. On the average, 181 ± 10 gait cycles were analyzed for each subject. Surface electromyography was recorded from 12 muscles of the dominant lower limb and the trunk. Gait cycles were grouped into subgroups containing 10 gait cycles each. The consistency of the muscle synergies extracted during the gait trial was assessed by measuring cosine similarity (*CS*) of muscle weights vectors, and zero-lag cross-correlation (*CC*) of activation signals. The average intra-subject *CS* and *CC* were 0.94 ± 0.10 and 0.96 ± 0.06, respectively. We found five synergies shared by all the subjects: high consistency values were found for these synergies (*CS =* 0.96 ± 0.05, *CC* = 0.97 ± 0.03). In addition, we found 10 subject-specific synergies. These synergies were less consistent (*CS* = 0.80 ± 0.20, *CC* = 0.89 ± 0.14). In conclusion, our results demonstrated that shared muscle synergies were highly consistent during walking. Subject-specific muscle synergies were also consistent, although to a lesser extent.

## Introduction

Human locomotion is a complex motor task, due to the many functions activated during gait cycles (Perry, 1992) and the multiple degrees of freedom of the skeletal muscle system (Bernstein, 1967). Previous research highlighted that the central nervous system (CNS) activates a small set of modules, called muscle synergies to control complex movements (Lacquaniti et al., 1999; Ivanenko et al., 2003; Cheung et al., 2005; Bizzi et al., 2008; Tresch and Jarc, 2009; Delis et al., 2010). According to the muscle synergies model, movements are produced by adapting a few activation patterns shared by several muscles. These patterns are generated by neural circuitry located in the lumbar spinal cord, which is responsible for producing the basic locomotor rhythm and allocating variable weights to different muscles (Ivanenko et al., 2004). Hence, complex tasks are actuated by basic activation patterns and the distribution of weights to the muscles (Zehr, 2005; Lacquaniti et al., 2012; Danner et al., 2015). The effectiveness of muscle synergies in modeling the complexity of motor control during gait has been demonstrated in several studies. Indeed, human gait can be described by a small set of robust synergies (Ivanenko et al., 2004; Monaco et al., 2010; Chvatal and Ting, 2012; Steele et al., 2015), while some other studies showed that different tasks can activate the same set of synergies (Chvatal and Ting, 2013). Repeatability of muscle synergies within and between days has been studied (Shuman et al., 2016). Moreover, a model of human locomotion encoding principle of legged mechanics was developed in Geyer and Herr (2010).

Muscle synergies during walking are generally extracted from a few gait cycles ranging from 1 (Steele et al., 2015; Lencioni et al., 2016) to 20 gait cycles (Kim et al., 2016). In most cases, synergies are computed by averaging or concatenating around 10 gait cycles (Ivanenko et al., 2003, 2004; Monaco et al., 2010; Chvatal and Ting, 2012; Coscia et al., 2015; Haghpanah et al., 2017). In spite of the cyclic and repetitive nature of walking, human gait is characterized by a high stride-to-stride variability of EMG patterns: to collect this variability, a large amount of gait cycles have to be recorded within each trial (Winter, 1987; Di Nardo et al., 2015; Rosati et al., 2017). It is reasonable to assume that, during a 5-min locomotion task, some motor adaptations may arise due to internal and external needs (e.g., undesired changes in gait speed, modified attention to the task, temporary proprioceptive, or sensory input changes, modified control of the task). This may or may not be reflected in consistent synergies during the task. However, since previous literature suggested that muscle synergies are cabled at a spinal level to provide a modular control of muscle groups during motor tasks (Saltiel et al., 2001; Dietz, 2003; Ting et al., 2015), we may hypothesize that muscle synergies are consistent during locomotion. However, it has not been fully investigated whether the motor control strategies remain consistent or change during a locomotion task. To fill this research gap, the present work aims at investigating the intra-subject consistency of muscle synergies during a 5-min walking trial. With “intra-subject consistency” of muscle synergies we refer to the specific subject showing synergies with similar muscle weights and activation signals along the whole trial. We also investigated which muscle synergies are “shared” between subjects, and, on the contrary, which are “subject-specific”, evaluating their respective degree of intra-subject consistency.

## Materials and Methods

### Subjects

Twelve young healthy females (age: 24.6 ± 1.6 years, height: 164.1 ± 6.8 cm, body mass: 54.1 ± 5.7 kg) were recruited for the study among the university student population. None of the subjects reported lower limb injuries or interventions, and none of them had neurological or musculoskeletal disorders that could compromise their gait. All of the subjects were right-limb-dominant according to the preferred foot to start the walking action (Sadeghi et al., 2000). This study involved healthy students in Biomedical Engineering from our university (Politecnico di Torino, Italy) for whom this was an experience useful also for their studies and it was exempt from Institutional Review Board approval given that there were no safety issues. The volunteers signed a written informed consent to participate in the study and the research reported in this paper was undertaken in compliance with the ethical principles of the Helsinki Declaration.

### Recording System and Signal Acquisition

A multichannel system for gait analysis (STEP32, Medical Technology, Italy) was used to acquire data. The system recorded: (1) surface electromyography (EMG) signals, (2) foot-switch signals, for timing the gait cycle, and (3) knee joint angle curves in the sagittal plane. Surface EMG signals were recorded by means of active probes (single differential configuration, size 19 mm × 17 mm × 7mm, 4-mm diameter Ag-disks, interelectrode distance 12 mm, CMRR over 126 dB), placed over 12 muscles of the dominant leg and trunk: vastus medialis (VM), tensor fasciae latae (TFL), gluteus medius (GMD), medial hamstring (MH), longissimus dorsii, at L4 level, right (LD_R_), and left (LD_L_), tibialis anterior (TA), lateral gastrocnemius (LGS), peroneus longus (PL), soleus (SOL), rectus femoris (RF), and lateral hamstring (LH). Foot-switch signals were acquired by three thin switches (size 10 mm × 10 mm × 0.5 mm; activation force: 3N) placed beneath the heel, the first, and fifth metatarsal-heads of each barefoot sole. Knee joint kinematics in the sagittal plane was collected, bilaterally, by electrogoniometers (accuracy: 0.5°) placed on the lateral side of each lower limb.

EMG signals were amplified to minimize, for each specific muscle, the quantization error; gain ranged from 60 to 86 dB. Data were acquired with a sampling frequency of 2000 Hz, converted by a 12-bit A/D converter and sent to a PC.

**FIGURE 1:**
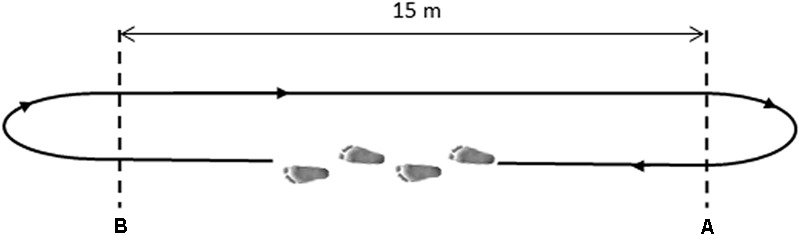
Schematic representation of the walking path. Subjects walked from point **(A)** to point **(B)** at their natural pace, then turned back and proceeded in the opposite direction.

After sensors positioning, subjects were asked to walk barefoot back and forth over a straight pathway of 15 m for 5 min (see **Figure 1**). Subjects were instructed to walk at self-pacing rhythm and to maintain it constant in the A–B tract. Before the recording session, the subject – equipped with all sensors – performed a 2-min preliminary trial to acquire confidence with the instrumented walking. Only gait cycles collected in the linear A–B tract were considered, removing gait cycles relative to direction changes, as described in the next section. The experimenter timed each subject’s passage through the A–B tract. The average gait speed was defined as the total distance walked in a straight line divided by the total time required going through it (Agostini et al., 2015).

### Signal Processing

The foot-switch signals were used to time the gait cycle. More specifically, foot-switch signals were debounced, converted to four levels [Heel contact (H), Flat foot contact (F), Push off (P), Swing (S)] and processed to segment and classify the different gait cycles (Agostini et al., 2013). Furthermore, only gait cycles consisting of the sequence of H–F–P–S were considered, discarding other possible non-standard cycles.

The knee joint angle signal was low-pass filtered (FIR filter, 100 taps, cut-off frequency equal to 15 Hz).

The knee range of motion and gait phases durations were exploited together to remove undesired strides relative to direction changes. In particular, strides involving curve negotiation, deceleration and acceleration before and after changing direction, were discarded by means of a multivariate statistical filter (Hotelling *t*-test, significance level α = 0.05) (Di Nardo et al., 2017).

### Muscle Synergies Extraction

Gait cycles were concatenated prior to filtering, with the aim of attenuating the cutting artifact (Gizzi et al., 2015). Indeed, in concatenating consecutive gait cycles it can happen that they are not also contiguous, e.g., when they are separated by deceleration/acceleration or by other outlier cycles.

The EMG of each specific muscle was concatenated considering 10 consecutive HFPS gait cycles. To study the consistency of muscle synergies, *N* subgroups of 10 gait cycles each were generated: subgroup 1 contained the EMG signal of HFPS gait cycles from 1 to 10, subgroup 2 from 11 to 20, and so on. The last subgroup was discarded if it contained less than 10 gait cycles. An EMG matrix *M*(*t*) (dimension *m* × *n*, where *m* was the number of muscles and *n* was the time points of 10 gait cycles) described each subgroup. The EMG signals were pre-processed before muscle synergies extraction. They were high-pass filtered at 35 Hz, demeaned, full-cycle rectified and low-pass filtered at 12 Hz by a 5th order Butterworth filter. Afterward, EMG of each channel was normalized in amplitude with respect to its global maximum, through the entire walk. The global maximum was calculated as the maximum RMS value of the signal over 50 ms time windows. Finally, the duration of each gait cycle was resampled into 1000 time points (Clark et al., 2009).

For each subgroup, muscle synergies were extracted with Non-negative matrix factorization (NMF) (Lee and Seung, 1999; Torres-Oviedo and Ting, 2007). NMF models muscular activity as a linear combination of muscular synergies activated by time-varying coefficients:

$$M(t)=\sum_{k=1}^{K}C{\left(t\right)}_{k}W_{k}+e$$

where *M*(*t*) is the EMG signal, *W*_k_ are the weights of the linear combination, *C*_k_(*t*) are the recruiting coefficients that vary in time, and *e* is the residual error. *W*_k_ defines the *k*-synergy (*k* = 1,…, *K*), whereas *C*_k_(*t*) expresses the neural signal that controls the *k*-synergy (Ting and Chvatal, 2010; Danner et al., 2015). The muscular activity estimated by NMF was compared with the original EMG signal using the Variance Account For (VAF) criterion. VAF expresses the amount of variation explicated by the model: the higher the VAF, the smaller the prediction error and, consequently, the better the model (Zar, 2010). Notice that, for each subgroup *i*, it may be required a different number of synergies *K*_i_ to accurately reconstruct the original signal. We chose the number of synergies *K*, common to all subgroups, in such a way as to obtain a VAF ≥ 90% for every subgroup. This requires calculating *K* as:

$$K\;=\;\max\left(K1,K2,\ldots ,K_{N}\right)$$

Since the NMF algorithm was applied to each subgroup of 10 gait cycles, *N* sets of *K* muscle synergies were obtained, one for each subgroup.

The *k*-means algorithm was adopted to order the synergies according to their weights *W*_k_ (Steele et al., 2015). The number of *k*-means classes was set equal to *K*. Clusters were randomly initialized and 10000 permutations, repeated five times, were performed. The coefficient matrices *C*_k_ were ordered correspondingly.

### Synergy Consistency

We evaluated the intra-subject consistency of muscle synergies by quantifying the similarity of muscle weights and activation signals among subgroups of 10 concatenated gait cycles.

For each synergy, we adopted cosine similarity (*CS*) as a metric of similarity between two weights vectors (D’Avella and Bizzi, 2005; Han and Kamber, 2007). The *CS* between two general subgroups *i* and *j* of a synergy *k* was computed as the normalized scalar product between the weights vectors

$$CS_{k}^{\mathrm{ij}}\;=\;\frac{W_{k}^{i}\cdot W_{k}^{j}}{∥W_{k}^{i}∥\;∥W_{k}^{j}∥}$$

where $W_{k}^{i}$ and $W_{k}^{j}$ are the vectors of weights of the i- and j-th subgroups, respectively. *CS* values range between 0 and 1 (0 = no similarity, 1 = complete similarity).

The degree of similarity of activation signals was computed as the value of the cross-correlation at zero time lag (*CC*) (Gizzi et al., 2011; Godlove et al., 2016). *CC* values range between –1 and 1.

### Shared Muscle Synergies

We selected shared synergies between subjects using the following criterion. We randomly chose a subject as reference and fixed on a specific weight vector. We calculated *CS* between the reference weight vector and all the vectors of another subject. The highest *CS* defined the first shared function of the two subjects. This procedure was iterated for each weight vector of the reference subject to define the other shared functions. We repeated the algorithm for every subject. Subsequently, an averaged set of similar muscle synergies for all subjects was computed (Torres-Oviedo and Ting, 2007; Hagio et al., 2015).

### Statistical Analysis

To analyze the intra-subject consistency of each muscle synergy, for each subject, we calculated the average *CS* and *CC* values and their standard error across subgroups of gait cycles. Since each subject showed a different number of synergies, no other specific statistical tests were performed on these data, at this stage.

For the selection of the shared synergies (between subjects), we averaged the weights and activation signals across subgroups. Then, we analyzed the differences, in consistency, among the shared muscle synergies. Both *CS* and *CC* data were tested for normality by means of a Kolmogorov–Smirnov test. Since both *CS* and *CC* data were not normally distributed, we applied a Kruskal–Wallis test to analyze the median differences among the shared muscle synergies. A *post hoc* Fisher Least Significant Difference (LSD) test was applied when appropriate.

Finally, for each subject-specific synergy we calculated the average and standard deviation of *CS* and *CC* values across subgroups. Since a subject (a) may not show any subject-specific synergy, (b) may show one or more subject-specific synergies, no further statistical analysis was performed on these data.

## Results

The 12 analyzed subjects walked at an average self-selected speed of 1.2 ± 0.1 m/s. On the average, 277 ± 11.5 gait cycles were recorded for each subject. After outlier removal, 181 ± 10 gait cycles were analyzed. As an example of EMG variability, we report onset/offset activation intervals of the tibialis anterior muscle of a representative subject relative to 163 gait cycles of her walking trial (Supplementary Figure S1). Furthermore, knee joint kinematics of each subject is reported in Supplementary Material (Supplementary Figure S2). For each subject, gait cycles were divided in 18 ± 1 subgroups of 10 concatenated gait cycles. Altogether, muscle synergies were extracted from 213 subgroups of 10 gait cycles.

### Number of Extracted Muscles Synergies and Analysis of Synergy Consistency

On the average 5.8 ± 0.6 muscle synergies were extracted for each subject. More specifically, five synergies were extracted in three subjects (VAF: 92.1% ± 0.6), six muscle synergies were extracted in eight subjects (VAF: 92.1% ± 0.3), and seven synergies were extracted in only one subject (VAF: 92.0%).

**FIGURE 2:**
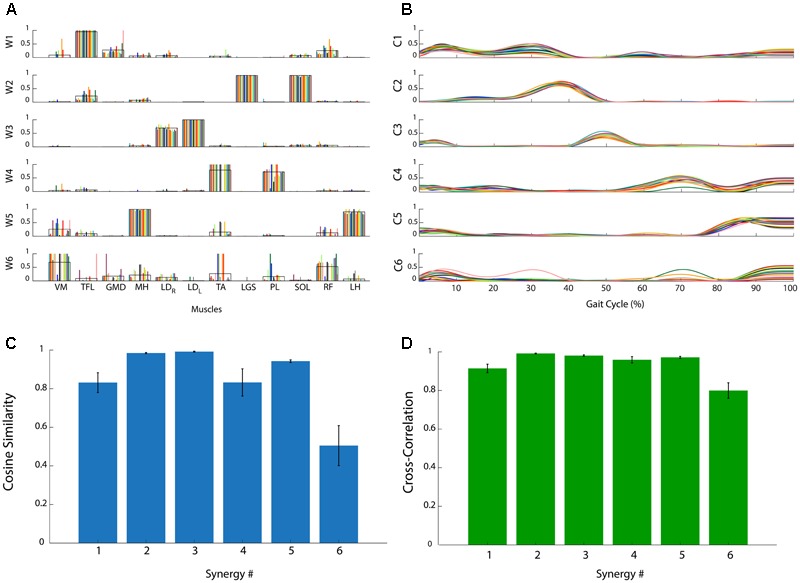
Analysis of the consistency of muscle synergies in a representative subject. **(A)** Muscle synergy weights (*W*_1_, …, *W*_6_): each colored bar represents a subgroup of 10 concatenated gait cycles. The black line represents the average across bars. **(B)** Muscle synergy coefficients (*C*_1_, …, *C*_6_): each colored line represents a subgroup of 10 concatenated gait cycles. **(C)** Cosine Similarity for the weights of each synergy. **(D)** Cross-Correlation coefficient for the activation signals of each synergy. Data are reported as mean ± SEM.

**Figure 2** reports the muscle synergies for a representative subject. The weights (**Figure 2A**) and coefficients (**Figure 2B**) of the muscle synergies are reported for each subgroup of 10 concatenated strides. It is evident that some synergies are very consistent among subgroups. As an example, synergy 2 is consistently dominated by muscles LGS, and SOL. In this case, these two muscles show a high value of weights (close to 1), similar among all subgroups, while the other muscles are scarcely represented (weights very close to zero). On the contrary, it can be observed that, in synergy 6, the contribution of the muscles is very variable. In some of the subgroups, weights are equal to 1 while in others they are zero. These observations are confirmed by the *CS* values reported in **Figure 2C**: *CS* is close to 1 in very consistent synergies (synergies 2, 3, and 5), while it decreases to 0.5 for the synergy 6 which is the least consistent. **Figure 2D** reports *CC* values for the activation signals. It can be observed that all activation signals are consistent across the task, with *CC* values above 0.8.

**FIGURE 3:**
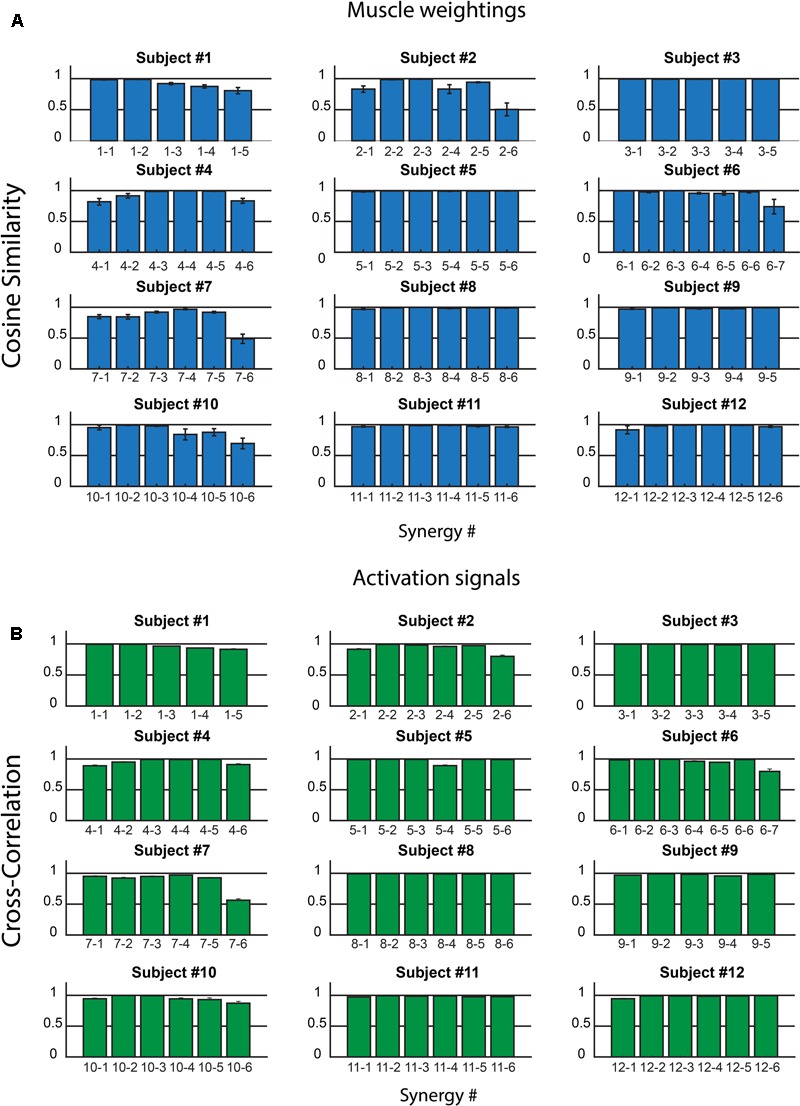
Intra-subject consistency of the muscle synergies in the 12 subjects: Cosine Similarity for the weights **(A)** and Cross-Correlation for the activation signals **(B)** are reported. In the *x*-axis labels, the first number identifies the subject, the second the synergy. Data are reported as mean ± SEM.

**Figure 3** shows *CS* (**Figure 3A**) and *CC* values (**Figure 3B**) for all the muscle synergies found in the twelve subjects. On the average, on all the synergies and all the subjects, *CS* was 0.94 ± 0.10, with values higher than 0.8 for most of the synergies. Only two synergies showed a *CS* lower than 0.5 (synergy 6, subject 2, and synergy 6 subject 7). Analogous results were found for *CC* and its average value was 0.96 ± 0.06.

### Shared Motor Functions and Their Muscle Synergies Consistency

A biomechanical function was assigned to each synergy by observing the prevailing muscles (weights > 0.5) and the profile of the coefficient curves (activation signals) (Winter, 1987; Perry, 1992).

**FIGURE 4:**
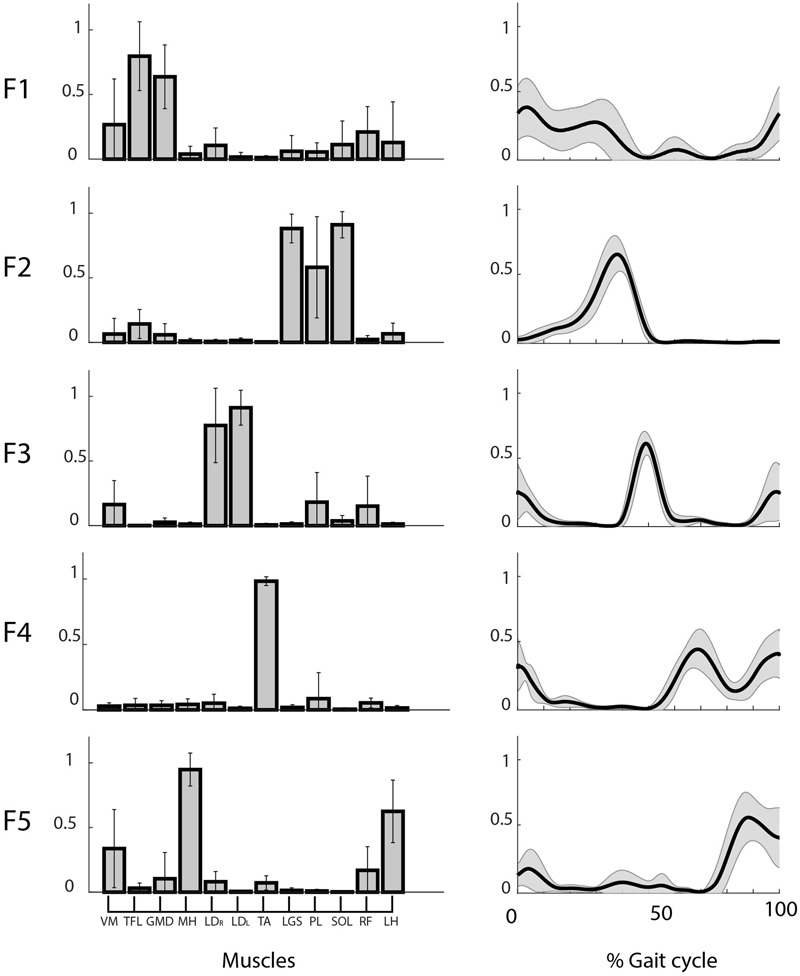
Weights (**left**) and coefficients (**right**) of the common muscle synergies across subjects. Muscle labels are reported below the weight plots. Data are reported as mean ± SD.

We found five motor functions common to all the subjects, and we labeled them from F1 to F5 (see **Table 1**). The muscle synergies accomplishing the same motor function were averaged among the twelve subjects (see **Figure 4**). Three motor functions were mainly related to the generation of the cyclic pattern of gait (F2, F4, F5), while two were related to body stabilization and dynamic balance control (F1, F3).

**Table 1:** Biomechanical functions of the shared muscle synergies during gait.

Synergy	Function	Principal muscles	Biomechanical function
1	F1	TFL – GMD	Stabilize hip joint during heel strike and the load acceptance phase
2	F2	LGS – PL – SOL	Generate propulsion at mid and terminal stance
3	F3	LD_R_ – LD_L_	Control the trunk position in the frontal plane at the heel strike of the homolateral and contralateral foot
4	F4	TA	Decelerate the foot during first rocker and control forefoot clearance during swing phase
5	F5	MH – LH	Decelerate the leg at the end of the swing phase

**FIGURE 5:**
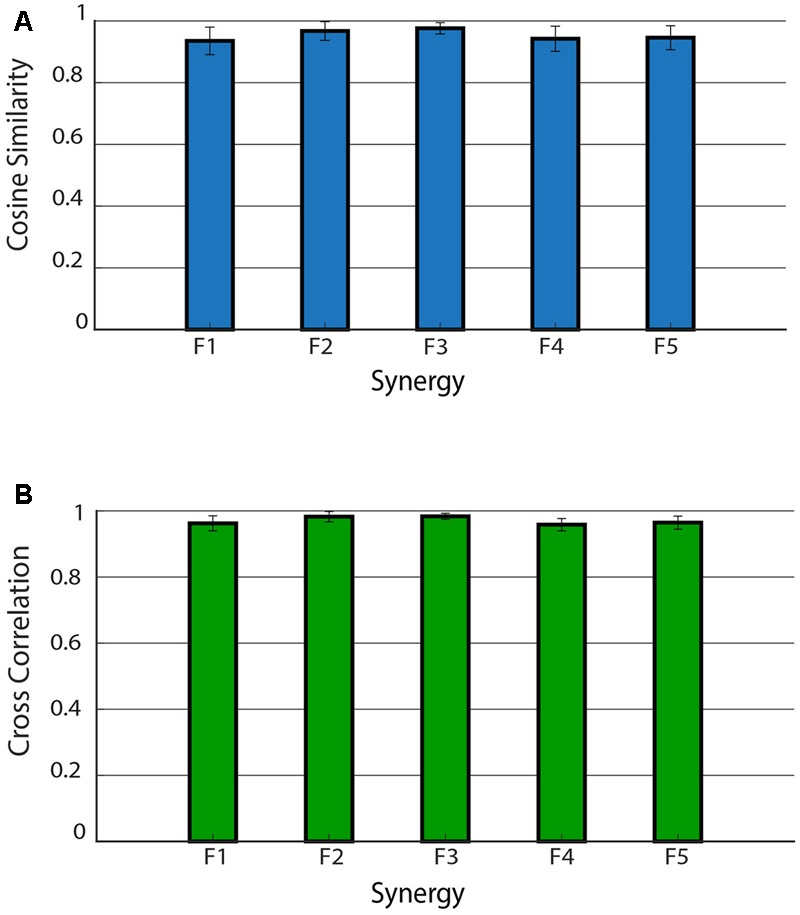
Cosine similarity for the weights **(A)** and cross-correlation for the activation signals **(B)** for the five shared muscle synergies. Data are reported as mean ± SD.

**Figure 5** shows the mean *CS* and *CC* values of the shared muscle synergies. It can be observed that all the values are above 0.9. No significant difference was found among *CS* values of different synergies (*p* = 0.17). A significant difference was found among *CC* values of different synergies (*p* = 0.03). The *post hoc* test showed that the *CC* median was significantly higher for F2 with respect to F1 and F4. This is confirmed by the results displayed in the right panel of **Figure 4** showing a smaller variability in the activation curves of F2 with respect to F1 and F4.

### Characteristic Subject-Specific Synergies

From 1 to 2 muscle synergies were characteristic of subjects. **Table 2** reports *CS, CC*, principal weights, and biomechanical functions of the subject-specific synergies. More specifically, we found subject-specific synergies in 9 out of 12 subjects: 8 subjects showed 1 subject-specific synergy, while 1 subject showed 2 subject-specific synergies. Overall, we found 10 subject-specific synergies, with an average *CS* equal to 0.80 ± 0.20 and *CC* equal to 0.89 ± 0.14. VM was activated in 6 out 10 synergies, followed by GMD (5 out 10), RF (3 out 10), and TFL, LGS, SOL, and (1 out 10). A motor function was assigned to each subject-specific synergy, similarly to the shared ones. The main motor functions aimed at decelerating leg at the heel strike and terminal swing phase.

**Table 2:** Intra-subject consistency of muscle weights and activation signals across subgroups of gait cycles, principal muscles recruited, and biomechanical functions of the subject-specific muscle synergies.

Subject	Consistency of motor functions		Principal muscles	Biomechanical function
	Muscle weights CS (mean ± SD)	Activation signals CC (mean ± SD)		
Subject #1	–	–	–	–
Subject #2	0.50 ± 0.22	0.80 ± 0.09	VM-RF	Stiff the knee at heel strike and the load acceptance phase
Subject #3	–	–	–	–
Subject #4	0.83 ± 0.08	0.91 ± 0.05	GMD-LGS-SOL	Stabilize hip joint and the foot at the heel strike
Subject #5	0.97 ± 0.01	0.99 ± 0.004	PL	Not defined
Subject #6	0.98 ± 0.03	0.99 ± 0.004	TFL-RF	Stabilize hip joint during swing
	0.74 ± 0.25	0.80 ± 0.17	VM	Not defined
Subject #7	0.49 ± 0.16	0.56 ± 0.09	GMD	Control the hip joint at heel strike and the end of the swing
Subject #8	0.99 ± 0.004	0.99 ± 0.004	VM-GMD-RF	Stiff the knee and control the hip joint at heel strike and the end of the swing
Subject #9	–	–	–	–
Subject #10	0.70 ± 0.17	0.87 ± 0.11	VM	Stiff the knee at heel strike and the end of the swing
Subject #11	0.96 ± 0.02	0.98 ± 0.01	VM-GMD	Stiff the knee and control the hip joint at heel strike and the end of the swing
Subject #12	0.97 ± 0.02	0.99 ± 0.01	VM-GMD	Stiff the knee and control the hip joint at heel strike and the end of the swing

*Subjects #1, #3, and #9 showed no subject-specific synergies, but only shared synergies.*

## Discussion

The present study evaluated, in young adults, the consistency of muscle synergies during a 5-min walking trial. The trial was divided into subgroups of 10 concatenated gait cycles each. Then, we extracted muscle synergies from each subgroup of gait cycles. To quantify muscle synergies consistency, we adopted the cosine similarity metric, for the weights, and the cross-correlation, for the activation signals.

### Methodological Observations

In our protocol design, natural pacing was preferred to constrained rhythm, to avoid influencing the synergies with biomechanical constraints (Steele et al., 2015). For the same reason, barefoot level walking was preferred to shoed treadmill walking (Sloot et al., 2014). The number of gait cycles within each subgroup was chosen to obtain a robust set of muscle synergies. It was demonstrated that, in healthy subjects, when calculated for small numbers of gait cycles the expected margin of error can change dramatically as a result of cycle-to-cycle variability. As the number of gait cycles increases, the margin of error decreases and stabilizes (Shuman et al., 2016). In addition, synergies extracted from 10 concatenated gait cycles are comparable with those obtained from more gait cycles (Oliveira et al., 2014).

### Five Motor Functions: Coherence with Literature and Biomechanics

Overall, we found 5 to 7 muscle synergies per subject. Among these, five were shared by the entire sample, in good agreement with previous studies. In fact, the number of synergies shared by healthy subjects can vary from four (Gizzi et al., 2012; Ranganathan and Krishnan, 2012; De Groote et al., 2014) to five (Ivanenko et al., 2004; Cappellini et al., 2006; McGowan et al., 2010), depending on the number and choice of muscles and the goodness of synergies criterion (Shuman et al., 2016). Each muscle synergy contributes to a precise biomechanical function (Routson et al., 2014). We found muscle synergies related to the hip joint stabilization (F1) (Zelik et al., 2014; Hagio et al., 2015), the terminal stance propulsion (F2), and the leg control during swing phase (F4 and F5) (Cappellini et al., 2006).

### Consistency of Shared and Subject-Specific Muscle Synergies

Shared muscle synergies had *CS* and *CC* values close to 1. These high values indicate a good consistency of these synergies over the entire walking trial. Shared synergies represent biomechanical tasks cyclically repeated. They represent motor control strategies that remain substantially consistent across many gait cycles. Indeed, the consistency of the five shared motor functions was the same when comparing the muscle weights, while a very slight difference was observed when comparing the activation patterns. In particular, the “propulsion” motor function F2 was found slightly more consistent with respect to the motor functions F1 and F4. This might suggest that activation signals, interpreted as supraspinal commands, may slightly change during the task, at least for some motor functions. Conversely, all the muscle weights, interpreted as basic motor modules fixed at a spinal level (Ting et al., 2015), seem to remain consistent across the task.

In addition to the shared synergies, one or two subject-specific synergies were also found in the majority of subjects, in accordance with previous works (Chvatal and Ting, 2012, 2013). Subject-specific synergies were mainly present during two demanding phases of gait, which are heel strike and terminal swing. Recruited muscles suggest that these synergies were devoted to maintaining balance, a critical end-point of motion control (Bauby and Kuo, 2000). In fact, due to dynamic balance demand, locomotion is characterized by a high variability of the hip and knee angular moments (Winter, 1995). Therefore, muscle synergies aimed at controlling the hip and knee joints may vary across subjects to optimize the balance task. This confirms the adaptability of muscle synergies, since they aim at adapting the global activity patterns to the kinetic and kinematic limb demands during locomotion (Ivanenko et al., 2004). In most cases, subject-specific synergies had *CS* and *CC* close to 1, like shared ones. Subject-specific synergies, like shared ones, may refer to motor tasks that subjects execute cyclically, and, consequently, they are mainly consistent during walking. It may be speculated that muscle synergy consistency reflects the contribution of lower motor neuron circuitries or the locomotion rhythmic signals produced by Central Pattern Generator (CPG) (Dimitrijevic et al., 1998). However, some subject-specific synergies had a *CS* and *CC* value consistently lower, while others had undefined motor functions. Specific sensory inputs may play a role in these synergies (Cheung et al., 2005). It was demonstrated that synergies can vary due to obstacle negotiation, speed transitions or specific motor tasks (Cheung et al., 2009; Chvatal and Ting, 2012; Hagio et al., 2015). Furthermore, some researchers demonstrated that muscle synergies are influenced by step-related sensory feedback and biomechanical events of the gait cycle (Ivanenko et al., 2003).

## Conclusion

In this study, we investigated the intra-subject consistency of muscle synergies during locomotion. We analyzed a sample of healthy young adults during a 5-min walking trial. Each subject showed at least five consistent synergies. These synergies were common between subjects and described the same motor functions. In addition, 9 out of 12 subjects showed also subject-specific synergies. Subject-specific muscle synergies were also consistent, although to a lesser extent.

Despite EMG variability of the single muscles, the neural control based on muscle synergies remains substantially unchanged over time (consistent). Muscle synergies are consistent because they control the cyclical execution of the locomotion task: this control does not change over time, although the single muscle execution (EMG) may change due to external or internal needs.

Future works will aim at investigating the subject-specific synergies, to clarify their origin and role in controlling human locomotion. This variability could be mainly influenced by low-level neuromechanical reactions.

## Author Contributions

DR, VA, and MK designed the study, collected the data, performed the analysis, and wrote the manuscript.

## Conflict of Interest Statement

The authors declare that the research was conducted in the absence of any commercial or financial relationships that could be construed as a potential conflict of interest.
