# Supplementary figures and images for: Intra-Subject Consistency during Locomotion: Similarity in Shared and Subject-Specific Muscle Synergies
Source: Front Hum Neurosci. 2017 Dec 4;11:586. doi: 10.3389/fnhum.2017.00586 (PMC5723022; doi:10.3389/fnhum.2017.00586)

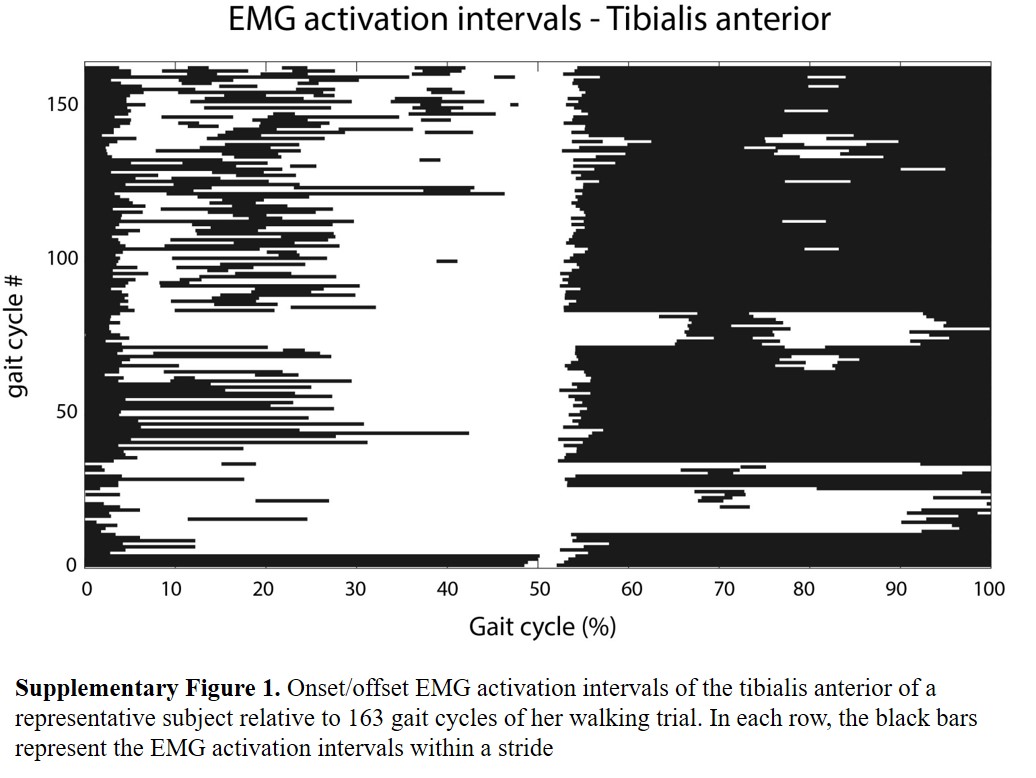

Supplement: Supplementary file 1 [file Image_1.JPEG]

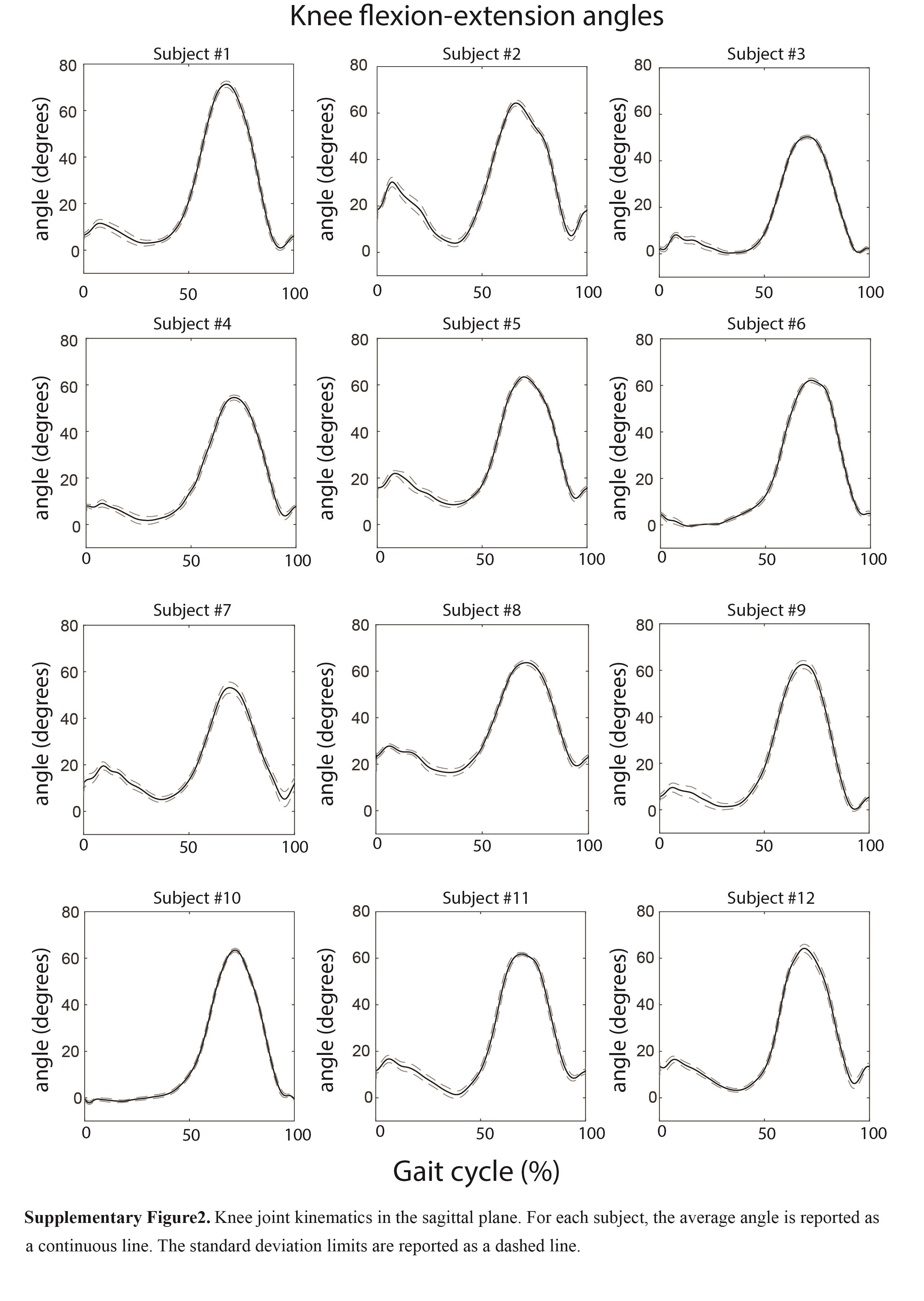

Supplement: Supplementary file 2 [file Image_2.JPEG]
